# Assessment of housing density, space allocation and social hierarchy of laboratory rats on behavioural measures of welfare

**DOI:** 10.1371/journal.pone.0185135

**Published:** 2017-09-19

**Authors:** Timothy Hugh Barker, Rebecca Peta George, Gordon Stanley Howarth, Alexandra Louise Whittaker

**Affiliations:** 1 School of Animal and Veterinary Sciences, The University of Adelaide, Roseworthy Campus, Wasleys, South Australia, Australia; 2 Gastroenterology Department, Children, Youth and Women’s Health Service, Adelaide, South Australia, Australia; Radboud University Medical Centre, NETHERLANDS

## Abstract

Minimum space allowances for laboratory rats are legislated based on weight and stocking rates, with the understanding that increased housing density encourages crowding stress. However, there is little evidence for these recommendations, especially when considering positive welfare outcomes. This study consisted of two experiments which investigated the effects of housing density (rats per cage), space allocation (surface area per rat) and social rank (dominance hierarchy) on the ability to perform simple behavioural tests. Male Sprague Dawley (SD) rats (n = 64) were allocated to either high-density (n = 8) or low-density (n = 8) cages. The second experiment investigated the effects of surface area. SD rats (n = 40) were housed in dyads in either the large (n = 10) or small (n = 10) cage. In both experiments, animals were tested on a judgment bias paradigm, with their responses to an ambiguous stimulus being ascribed as optimistic or pessimistic. Animals were also tested on open-field, novel-object recognition and social-interaction tests. Recordings were taken from 1700-2100h daily for rat observation and social rank establishment. Dominant animals responded with significantly more optimistic decisions compared to subordinates for both the housing density (p<0.001) and space allocation (p = 0.0015) experiment. Dominant animals responded with increased social affiliative behaviours in the social-interaction test, and spent more time in the centre of the open-field test for both experiments. No significance was detected between housing density or space allocation treatments. These findings suggest that social rank is a significantly greater modifier of affective state than either housing density or space allocation. This finding has not yet been reported and suggests that future drafts of housing guidelines should consider animal social status in addition to floor space requirements.

## Introduction

International standards for the care and housing of Lab Animals provide relatively uniform guidelines regarding stocking rates and surface area allowance afforded to all rodents used for scientific purposes. The *Guide for the Care and Use of Lab Animals* [1] (United States) and the *EC Directive 2010/63/EU* [2] (Europe) are among a few of the regulatory bodies that provide guidelines for rodent housing. These guidelines usually state specific measurements regarding the weight of the animal and the floor area allocated per animal to reduce the effects of crowding-related stressors. However, it has been recently noted that these guidelines rarely cite scientific literature to support these space requirements [3]. It has also been noted in the *Resolution on Accommodation and Care of Lab Animals* [4] that evidence-based data is lacking on this specific subject. While the scientific community have identified this knowledge gap, recent literature has focussed primarily on mice [5]. Few publications have identified the need to establish the effects of housing density and space allocation in rats (*Rattus norvegicus)*.

Investigations of space use by rats have been historically well-researched, however the primary focus was conducted in wild rats [6, 7]. Cage floor area and space allocation provide logistical limitations to the types of research activities that can occur [5] which perhaps prompted early research methods to study the effects that crowding can elicit [8]. It was first noted by Calhoun [8] that when a population of laboratory rats was allowed to increase in a confined space, abnormal behavioural patterns began to occur. It was argued that these behaviours could lead to the extinction of the entire caged population. This crowding effect has led to the prominence of guidelines and legislative documentation that encourage strict space allowances for each caged animal. However, this may not be an accurate portrayal of the multitude of factors that interact. Housing density is defined hereafter as the number of animals that occupy the same caged floor area while space allocation is defined as the surface area allocated to each animal within a shared cage. Housing density and space allocation are two separate, yet closely linked factors that interact to produce ‘crowding’. Crowding is the operational word used by these regulatory bodies that defines the motivational state that occurs when spatial and social factors interact [9]. Many studies that have previously discussed the effects of housing density or space allocation on rodent behaviour have been confounded by their inability to successfully separate these two factors [10, 11]. Studies in which cage size is kept constant and animals are added or subtracted are not designed to investigate either housing density or space allocation, instead they report the effects of crowding.

We sought to assess the effects of both housing density and space allocation on the performance of rats in an array of simple behavioural tests. We also aimed to determine if the social class (as determined through a dominance hierarchy) of the animals would interact with these factors, as this interaction had been significantly underreported in the literature, despite the knowledge of social composition contributing to crowding stress [9]. The behavioural tests utilised included the open-field test (OFT), the novel-object recognition test (NORT), the social-interaction test (SIT) and cognitive bias detection through a judgment bias paradigm (JBP). These tests were chosen due to their repeatability and their reliability at providing evidence of anxiety-like behaviours. The JBP has the added advantage of being able to identify positive welfare outcomes.

## Materials and methods

### Animals and housing

This study was separated into two distinct experiments. The first studied the effects of housing density and used 64 male Hsd: Sprague Dawley (SD) rats. The second study studied the effects of space allocation and used 40 male SD rats. All animals were sourced from a barrier-maintained, specific pathogen free production facility (University of Adelaide, Laboratory Animal Services, Adelaide, Australia). Upon arrival at the testing facility, animals were housed in their treatment groups (discussed later) in commercially available cages (Tecniplast, Exton, PA, USA). Cage design and specifications are discussed below. Each cage was provided with a paper-based bedding substrate (Animal Bedding, Fibrecycle Pty Ltd, Yatala, Queensland, Australia). Standard rat chow (Rat and Mouse Cubes, Speciality Feeds, Western Australia, Australia) and reverse-osmosis water were provided *ad libitum*. Room temperature was maintained between 21°C and 23°C and a reversed 12-hour light/dark cycle (lights on at 1800h, off at 0600h) was used. The animals were acclimatized to the facility environment for 5 days before behavioural training on the judgment bias paradigm commenced. All animal use and housing protocols were approved by the Animal Ethics Committee of the University of Adelaide and conducted in accordance with the provisions of the *Australian Code for the Care and Use of Animals for Scientific Purposes [12]*.

### Housing density experiment

**Fig 1:**
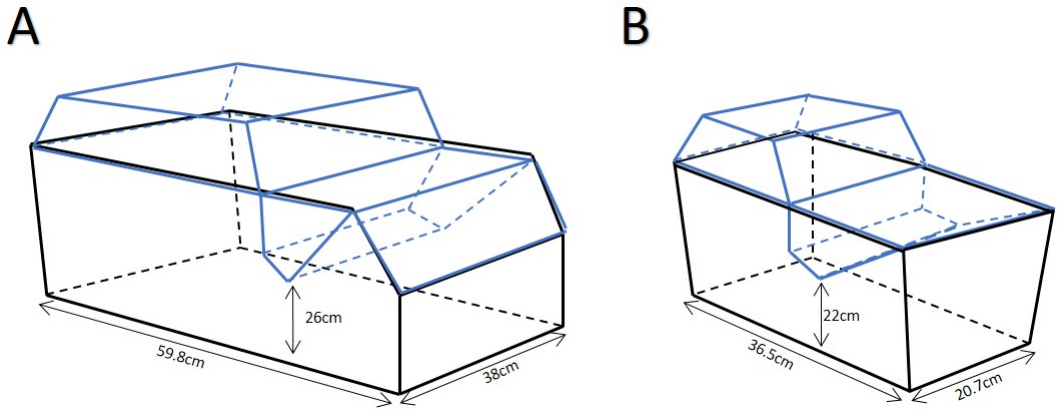
Diagram of cage sizes used. A) The Eurostandard type IV cage drawn to approximate 1:10 scale. This cage is appropriate to house up to 6 rats of approximately 450 grams. B) The Eurostandard type IIL cage drawn to approximate1:10 scale. This cage is appropriate to house up to 2 rats of approximately 450 grams. The black lines represent the cage bottom while the blue lines represent the cage lid.

Upon arrival at the testing facility, rats (n = 64) were housed in groups of either 6 (high-density), or 2 (low-density) rats per cage. The high-density cages (n = 8, total of 48 rats) were the Eurostandard type IV (Tecniplast, Exton, PA, USA) with dimensions of 59.8cm by 38cm with 26cm of vertical space at the lowest point (Fig 1A). These cages had an area of 2,280cm^2^ and were appropriate to house 6 rats of approximately 450 grams, per the guidelines as prescribed in the eighth edition of the *Guide for the Care and Use of Lab Animals* [1]. Low-density cages (n = 8, total of 16 rats) were the Eurostandard type IIL (Tecniplast, Exton, PA, USA) with dimensions of 36.5cm by 20.7cm with 22cm of vertical space at the lowest point (Fig 1B). These cages had an area of 755.55cm^2^ and were appropriate to house 2 rats up to 450 grams.

### Space allocation experiment

The second experiment used 40 male SD rats and occurred immediately following the completion of all behavioural testing of the housing density experiment. Animals were randomly housed in either the large cage (Eurostandard type IV) or the small cage (Eurostandard type IIL) with one other conspecific and allowed to acclimatize to these conditions for 3 weeks. Animals housed in the large cages were the large surface area treatment group (n = 10, total of 20 rats) and those housed in the small cages were the small surface area treatment group (n = 10, total of 20 rats).

### Cognitive bias test

**Fig 2:**
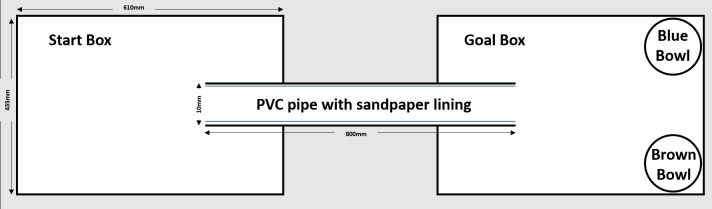
Diagram and size of judgment bias testing apparatus utilized. The judgment bias test was comprised of two Perspex boxes connected via a PVC pipe. Two reward bowls were placed in either corner of the goal box containing scented sand.

Cognitive bias detection has been used as an indicator of animal affective state (emotional state) [13]. These biases were measured using a judgment bias paradigm (JBP), that was based on an earlier JBP design [14]. Commonly used terminology of the JBP has been defined in Table 1. This JBP makes use of a single testing/training apparatus that comprises two Perspex boxes (610mm x 435mm x 500mm) connected via a PVC pipe with a 100mm diameter. During certain training phases, the pipe was lined with one of two-grades of sandpaper, one being coarse (P80) the other being a fine sandpaper (P1200). Inside one box (henceforth referred as the ‘goal box’) were two reward bowls positioned in the two far corners. These reward bowls were filled with either a coriander or cinnamon scented sand (1% by weight of spice to sifted sand). The coriander scented reward bowl remained in the right-hand corner for each trial, while the cinnamon scented reward bowl remained in the left-hand corner (Fig 2). Milk chocolate baking chips (Cadbury, London, England) were used as the high-positive reward items whilst Cheerios (Uncle Toby’s, Victoria, Australia) were considered a low-positive reward items. Every animal was randomly assigned a sandpaper association to the reward items and rewarded location for both the housing density experiment (Table 2) and the space allocation experiment (Table 3). This paradigm has been divided into phases where different experimental outcomes are expected. A summary of these phases is included in Table 4. The animals would learn to associate the different type of sandpaper with the type of reward item and where that reward item was located. During the testing phase, the sandpaper in the PVC pipe was replaced with sandpaper of an intermediate grade (P180) and no reward items were present in the reward bowls. This sandpaper type behaved as the intermediate, ambiguous probe and the responses to this probe could be considered either optimistic or pessimistic. An optimistic decision was defined when the rat displayed foraging behaviours for these intermediate ambiguous trials in the bowl that would normally contain the chocolate reward. A pessimistic behaviour was defined when the rat displayed foraging behaviour in the bowl that would normally contain the cheerio reward [15]. Testing in the JBP occurred once a day, for 5 days (Table 4).

**Table 1:** Definitions of commonly used terminology for the judgment bias paradigm.

Term	Definition
**Approach**	When the rat actively and intentionally placed its forelimbs and face into a reward bowl to extract the reward.
**Forage**	When the rat continuously and deliberately displaced the sand in the food bowl to obtain the reward.
**Consumption**	When the rat actively and intentionally interacted with the food by bringing it to its mouth.
**Success**	Successful trial was determined after the animal had approached and foraged in the correct (reward containing) food bowl before approaching or foraging in the incorrect food bowl.
**Promotion**	Animals were promoted to the succeeding trial (where appropriate) after achieving ¾ successful trails per day, for 5 consecutive days.
**Failure**	If the rat failed to consume the reward within 10 minutes of being placed into the testing chamber.

First described by Barker et al. (15)

**Table 2:** Associations of reward items and locations for treatments of the housing density experiment.

	Cage Density	Chocolate Stimulus	Chocolate Location	Cheerio Stimulus	Cheerio Location
**Association 1 (n = 12)**	High	Coarse Sandpaper	Brown Bowl / Right	Fine Sandpaper	Blue Bowl / Left
**Association 2 (n = 12)**	High	Coarse Sandpaper	Blue Bowl / Left	Fine Sandpaper	Brown Bowl / Right
**Association 3 (n = 12)**	High	Fine Sandpaper	Brown Bowl / Right	Coarse Sandpaper	Blue Bowl / Left
**Association 4 (n = 12)**	High	Fine Sandpaper	Blue Bowl / Left	Coarse Sandpaper	Brown Bowl / Right
**Association 5 (n = 4)**	Low	Coarse Sandpaper	Brown Bowl / Right	Fine Sandpaper	Blue Bowl / Left
**Association 6 (n = 4)**	Low	Coarse Sandpaper	Blue Bowl / Left	Fine Sandpaper	Brown Bowl / Right
**Association 7 (n = 4)**	Low	Fine Sandpaper	Brown Bowl / Right	Coarse Sandpaper	Blue Bowl / Left
**Association 8 (n = 4)**	Low	Fine Sandpaper	Blue Bowl / Left	Coarse Sandpaper	Brown Bowl / Right

Each association was randomly assigned and counter-balanced between treatments.

**Table 3:** Associations of reward items and locations for treatments of the space allocation experiment.

	Cage Size	Chocolate Stimulus	Chocolate Location	Cheerio Stimulus	Cheerio Location
**Association 1 (n = 5)**	Large	Coarse Sandpaper	Brown Bowl / Right	Fine Sandpaper	Blue Bowl / Left
**Association 2 (n = 5)**	Large	Coarse Sandpaper	Blue Bowl / Left	Fine Sandpaper	Brown Bowl / Right
**Association 3 (n = 5)**	Large	Fine Sandpaper	Brown Bowl / Right	Coarse Sandpaper	Blue Bowl / Left
**Association 4 (n = 5)**	Large	Fine Sandpaper	Blue Bowl / Left	Coarse Sandpaper	Brown Bowl / Right
**Association 5 (n = 5)**	Small	Coarse Sandpaper	Brown Bowl / Right	Fine Sandpaper	Blue Bowl / Left
**Association 6 (n = 5)**	Small	Coarse Sandpaper	Blue Bowl / Left	Fine Sandpaper	Brown Bowl / Right
**Association 7 (n = 5)**	Small	Fine Sandpaper	Brown Bowl / Right	Coarse Sandpaper	Blue Bowl / Left
**Association 8 (n = 5)**	Small	Fine Sandpaper	Blue Bowl / Left	Coarse Sandpaper	Brown Bowl / Right

Each association was randomly assigned and counter-balanced between treatments.

**Table 4:** Descriptions and promotion criteria of each training and testing phase involved in the JBP.

Phase	Description	Promotion Condition
**A**	Rats handled for two 10-minute periods. The first period between 0900 and 1200 hours, the second period between 1400 and 1700 hours.	Phase lasted for 5 days.
**B**	Rats placed into the testing apparatus, four times a day for 5-minute intervals. The food bowls contained the reward items appropriate to the individual rat, these rewards were placed on the surface of the sand in the reward bowls. No sandpaper was present within the PCV pipe.	Phase lasted for 5 days.
**C**	The testing apparatus now contained the appropriate sandpaper stimuli. Animals had two training trials between 0900-1200h and two between 1400–1700 hours. Each period had one chocolate trial and one cheerio trial that occurred in a random order. For each trial, a single reward item was placed on the surface of the appropriate reward bowl, to the appropriate sandpaper that was present in the apparatus. Rats were placed in the start box, upon which a timer was started. Latency for the rat to leave the start box, enter the goal box, approach any reward bowl, approach the correct reward bowl and start to consume the reward was recorded. The rat was immediately removed from the apparatus once it had consumed the reward or if it failed the test. The whole apparatus was then cleaned with 70% ethanol solution.	Promotion to phase D was achieved after the animals achieved success on 3 of their 4 daily trials for once a day, for 5 days in a row.
**D**	Identical to phase C, however during phase D the reward items were buried in the sand of the reward bowls. Each rat was required to forage for the reward item and extract it from the sand. Following the successful extraction of the reward, the depth at which the reward was buried for the next trial increased. Burial depth continued to increase with each successive trial until the reward was always completely buried in the sand.	Promotion to phase E was identical to the promotion conditions of phase C
**E**	Identical to phase D, but the reward items were always completely buried in the sand and one randomly selected trial per day contained no reward item. A successful, unrewarded trial was defined when the first bowl that the rat foraged in would normally contain a reward item.	Promotion to phase E was identical to the promotion conditions of phase C
**F**	Identical to phase E, except the unrewarded trial was now paired with a sandpaper of intermediate grade (P180).	Phase lasted for 3 days
**G**	Testing Phase. The rats received one test per day that involved the intermediate sandpaper (P180) being present in the pipe and no reward being present in the food bowl. During testing, the time was recorded for the rat to forage in any bowl, and the bowl the rat approached, and foraged in first was documented.	Phase lasted for 5 days

Adapted from Brydges *et al*. (14)

### Open-field test

**Fig 3:**
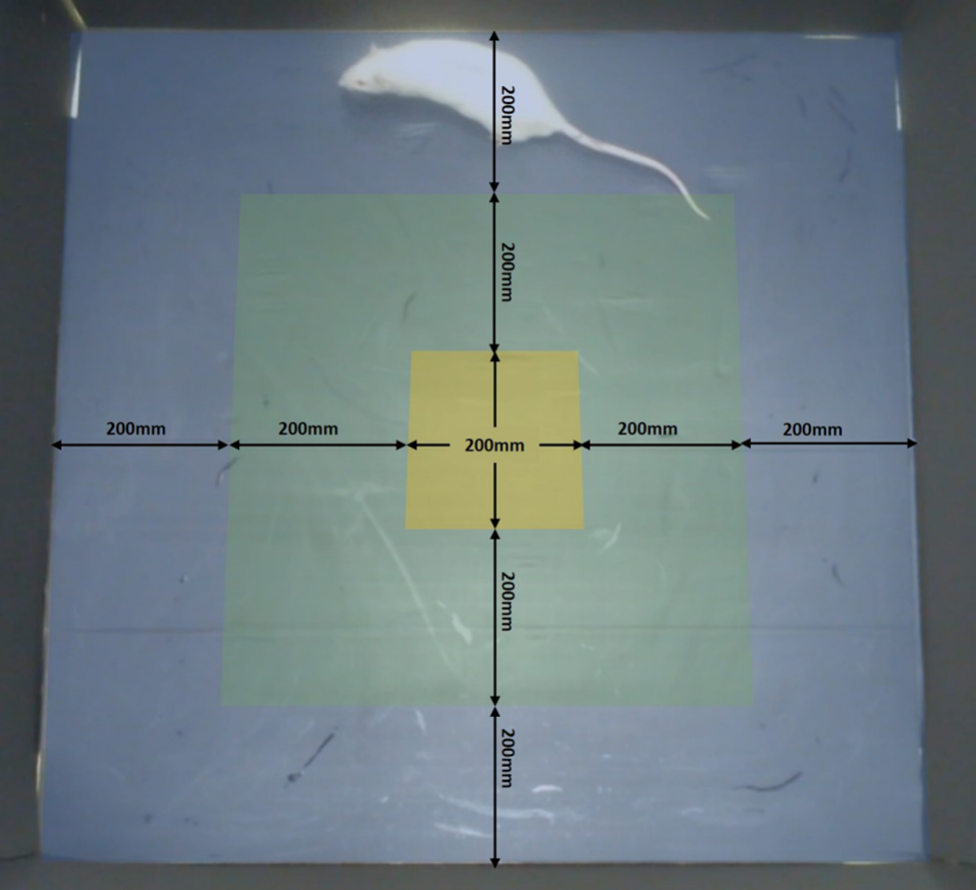
Diagram of the open-field test. Image of the video recording of the open-field test detailing the location and size of the zones used. Blue: Peripheral Zone, Green: Inner Zone, Yellow: Centre Zone. Arrows have been superimposed to indicate size.

On day 2 of the five-day testing period, each animal was subjected to the open-field test (OFT) after recording a cognitive bias decision for that day. Testing was performed as described by the methods of Wallace [16] and utilised a square testing arena (100cm by 100cm by 100cm) made from black corflute in a homogenously illuminated arena (150 lux) away from where the animals were normally housed. Animals were placed individually into the centre of the arena upon which a video camera (Logitech HD Webcam C525, Lausanne, Switzerland) suspended over the centre of the arena began recording. The experimenter then immediately left the room and the animals stayed in the arena for 10 minutes. After 10 minutes the animal was removed from the arena and placed back into its home cage. The arena was then cleaned with a 70% ethanol solution. OFT video analysis was conducted manually utilising the CowLog open source software. Three zones were superimposed to the open field test video files (Fig 3) and the time spent in each zone was recorded, as was the number of transitions into each zone. Observers also recorded the time spent inactive during the test, time spent rearing, and number of defecation or urination incidents. A rat was considered to have entered a ‘zone’ when its centre of gravity had crossed into the new zone.

### Novel-object recognition test

**Fig 4:**
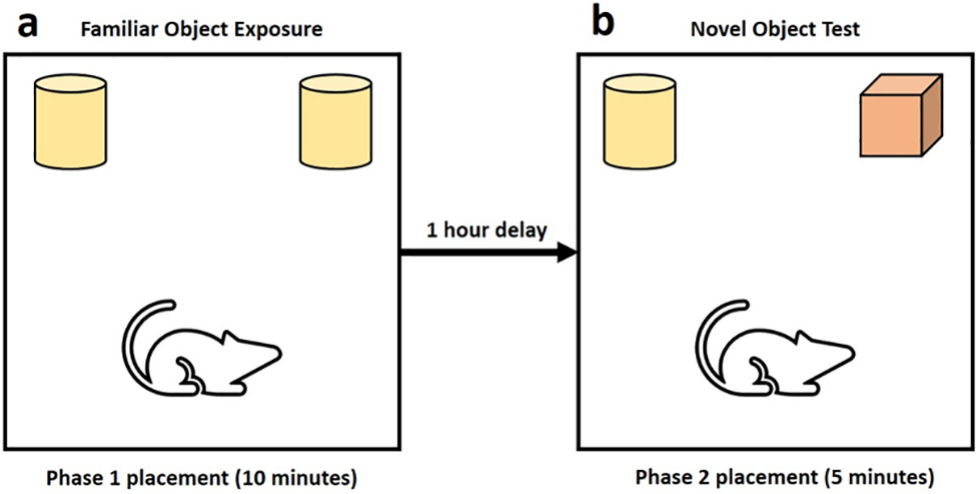
Novel object recognition test design. (A) Phase 1: Familiar object exposure. (B) Phase 2, Novel-object recognition test. From the methods of Bevins and Besheer [17]

On the third day of testing, after recording a cognitive bias result, each animal was subjected to the novel-object recognition test (NORT). Testing was performed according to the methods presented by Bevins and Besheer [17], the arena utilised was a barren (no bedding), ‘high-density’ home cage, with accompanying wire-lid. Testing involved two behavioural phases, during phase 1 (Fig 4A) an individual animal was placed into this testing arena with two identical objects (stainless steel, water bottle tops) in either corner. The experimenter than left the room after starting the video recording from the suspended camera. After 10 minutes, the animal was removed from the arena and placed back into the home cage and the arena was cleaned with a 70% ethanol solution. After one hour, the animal was ready to be tested again in the same arena, however during phase 2 (Fig 4B) one of the two familiar items from phase 1, was randomly replaced with a novel item (red, large die), of approximate equal mass to the familiar item. After the animal was placed into the testing arena for phase 2, the video recording was again started and the experimenter left the room for 5 minutes. After 5 minutes, the animal was placed back into the home cage and the arena was cleaned with a 70% ethanol solution. Video footage from phase 2 was used to analyse the NORT data. Using the CowLog open source software, the observer documented the time each rat spent interacting with both the familiar and the novel object, as well as the number of interactions that occurred for each object. Other measures taken include the time the animal spent exploring the confines of the arena, time spent rearing and inactive, and the number of defecation and urination incidents.

### Social-interaction test

On day 4 of the five-day testing period, each animal was subjected to the social-interaction test (SIT) after they had recorded a cognitive bias decision for that day. Testing was slightly modified from the methods of Sams-Dodd [18], and was performed in a circular arena with a diameter of 100cm made from hessian supported by flexible plastic sheeting. The arena was in a homogenously illuminated (150 lux) area in a different room to where the animals were normally housed. Test animals was marked with a nontoxic black marker immediately prior to being placed in the arena. The test animal was placed simultaneously into the arena with an unfamiliar SD rat (non-cage mate) that was not used as part of this study. This animal will henceforth be referred as the unfamiliar rat. Both test and unfamiliar rats were placed approximately 40cm apart in the arena. The video camera suspended over the arena began recording and the experimenter then left the room. Testing lasted for 10 minutes after which time the experimenter re-entered the room and returned each animal to its appropriate home cage before cleaning the arena with a 70% ethanol solution. Video analysis was performed manually with the CowLog open source software, animal behaviour was scored using a continuous sampling method. Behavioural expression was categorised using the ethogram as prescribed in Sams-Dodd [18] (Table 5). The time spent exhibiting each behaviour was totalled and a time-budget was generated. Each behavioural expression is presented as a percentage of the total time exhibited from the time-budget.

**Table 5:** Ethogram used for behavioural analysis during the social interaction test.

Behaviour	Description
**Exploration**	Movement in the arena, sniffing at floor, walls and inspecting arena.
**Rearing**	Raised on the hind legs, sniffing into the air.
**Investigation**	Sniffs at and investigates the unfamiliar rat.
**Follow**	Follows the unfamiliar rat.
**Grooming**	Cleaning the fur and/or scratching.
**Inactive**	No discernible action.
**Stationary Stereotyped Behaviour**	Stationary and performs circular head movements and/or head weaving.
**Lateral Threat**	Body is arched in a sideward posture towards the unfamiliar rat.
**Upright**	Standing on its hind legs and is facing the unfamiliar rat.
**Stand Over**	Standing on top of the unfamiliar rat.
**Lie Under**	Lying on its back beneath the unfamiliar rat.
**Clinch**	Active fighting with the unfamiliar rat.
**Pursue**	Runs after the unfamiliar rat during “Clinch”
**Escape**	Runs away from the unfamiliar rat during “Clinch”

Adapted from Sams-Dodd (18)

### Social classification

Classification of the rats into their social classes was achieved through observing video footage recorded with CCTV cameras (OzSpy, Brisbane, Australia). Rats were recorded from 1500-2300h each day over the five day testing period. Each animal was observed for 10 minutes with the start time of each sample being randomly selected between 1500-1750h. Behaviours of each animal were scored per the ethogram as originally devised by Hurst et al. (1996) (Table 6.). Interactions between each individual with respect to each of its caged conspecifics were examined to determine the dominance relationship. Total numbers of aggressive encounters initiated were summed and compared against the number of aggressive encounters received, to assign the animal an agonistic score. As per the experimental design of Hurst et al. [19], dominance within a dyad was assigned if the animal initiated greater numbers of aggressive encounters than it received over each of the five 10-minute videos. Social class of rats for the housing density experiment is included in Table 7, and social class of rats for the space allocation experiment is included in Table 8.

**Table 6:** Ethogram used for behavioural analysis during social classification.

Behavioural category	Behavioural elements of the viewed rat
**Sleeping**	Lying or sitting unalert, eyes closed
**Feeding/drinking**	Eating food or faeces; drinking
**Non-intake maintenance**	Grooming; yawning; stretching; sneezing; urinating; defecating
**Exploration**	Sniffing air, floor, wall, water bottle, faeces, urine or bedding
**Stationary**	Alert (eyes open) but no directed attention while lying, sitting or leaning
**Movement**	Alert but no directed attention while walking, stretching, climbing or running
**Other non-social behaviour**	Chewing bedding; digging/scrabbling; jumping
**Aggressive action**	Bite; chase; aggressive over (pinning rat on its back); aggressive groom; aggressive sideways; upright; mounting; pull tail, pursuit of fleeing rat
**Defensive action**	Defensive over (on back, being pinned), defensive sideways, flight (with and without pursuit)
**Social investigation**	Sniffing nose, mouth, head, shoulders, back, flank, anogenital area, belly, tail
**Other social behaviour**	Attend; allogroom

Originally designed by Hurst *et al*. (19) This ethogram was used to assess social hierarchy within cages and to assign dominance to animals within dyads.

**Table 7:** Social classes of caged rats for the housing density experiment.

Social Class	Definition	No.
**(D) Dominant**	Dominant over all cage mates–dominant in every dyad	n = 16
**(DS) Dominant subdominant**	Mostly dominant–dominant in most dyads but not all	n = 14
**(SS) Subordinate subdominant**	Mostly subordinate–subordinate in most dyads but not all	n = 22
**(S) Subordinate**	Subordinate to all cage mates–subordinate in every dyad	n = 12

Definitions of each class and criteria for assignment into a social class for the housing density experiment. As designed by Hurst *et al*.(19).

**Table 8:** Social classes of caged rats for the space allocation experiment.

Social Class	Definition	No.
**(D) Dominant**	Dominant over conspecific	n = 20
**(S) Subordinate**	Subordinate to conspecific	n = 20

Definitions of each class and criteria for assignment into a social class for the space allocation experiment. As designed by Hurst *et al*.(19).

### Statistical analysis

All data were analysed using the IBM SPSS Statistics 22 (IBM, NY, USA) software package. Levene’s test was used in all cases to test for normality of the data set, all data were found to be parametric unless otherwise stated. Numerical data have been presented as mean ± standard error of the mean. Differences between means were considered significant when *p* was less than 0.05. All normally distributed data was analysed using a two-way multivariate analysis of variance (MANOVA) test, fitting housing density and social class on the test variable. Where the data were not normally distributed, they were analysed using the Kruskal-Wallis H test and/or the Mann-Whitney U test. Further details of statistical analysis are found in the results section.

**Fig 5:**
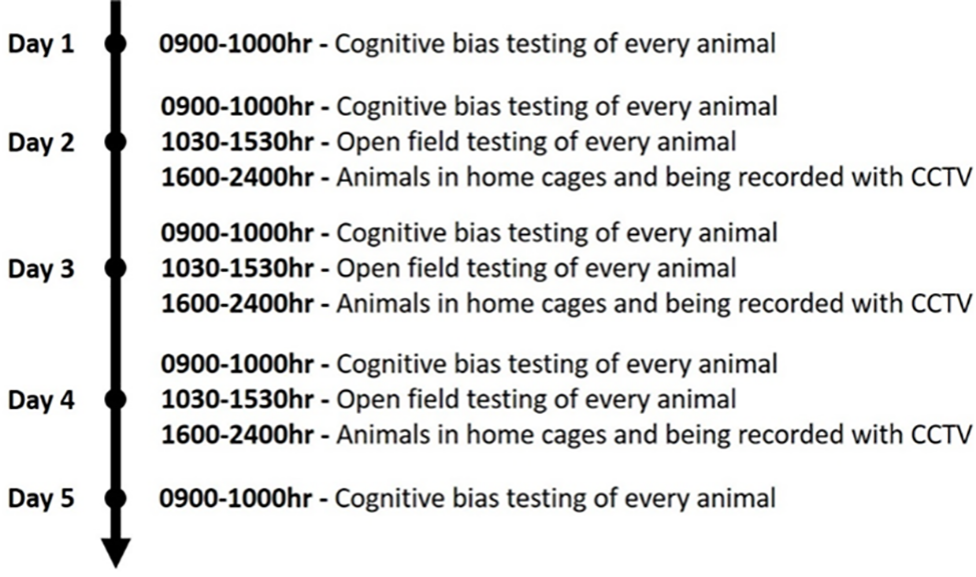
Flowchart of the experimental procedure and the days and times tests are performed. This flowchart details the steps used for both the housing density and the space allocation experiment. Despite the protocol remaining the same, it is important to note that these did not occur at the same time. The space allocation experiment occurred after the animals in the housing density experiment finished testing.

A complete flowchart of the experimental procedure is illustrated in Fig 5.

## Results

### Effects of housing density and social class on the cognitive bias test

**Fig 6:**
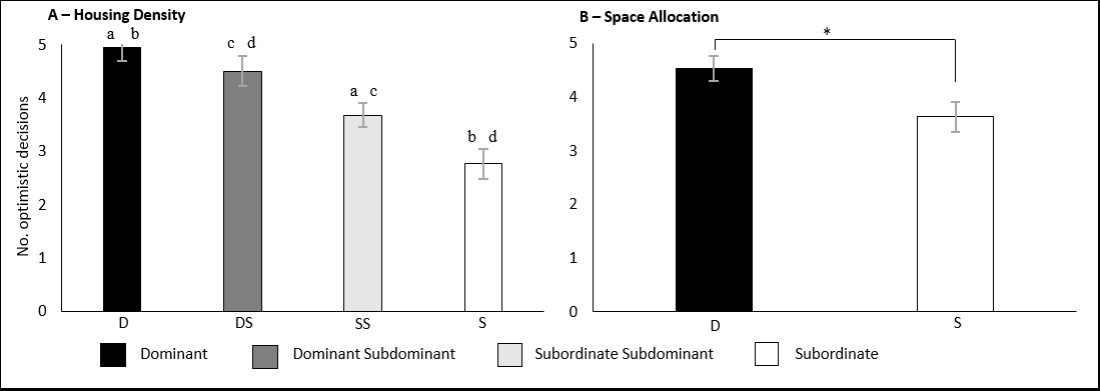
The effects of social class on the number of optimistic responses made in the JBP. A) For the housing density experiment. B) For the space allocation experiment. Significance is denoted at p< 0.05.

The Shapiro-Wilk test determined that the data were not normally distributed. A Kruskal-Wallis H test showed that there was no significant effect or interaction involving housing density on the number of days featuring an optimistic decision χ2(3) = 0.082, p = 0.521. However, there was a statistically significant difference observed in social class on number of optimistic decisions made, χ^2^(3) = 26.95, p<0.001. The Mann-Whitney U test was used to investigate the nature of this effect. Dominant (D) animals responded with significantly greater number of optimistic decisions (4.94 ± 0.25) compared to both Subordinate Subdominant (SS) animals (3.68 ± 0.22; p<0.001) and Subordinate (S) animals (2.77 ± 0.28; p<0.001). Likewise, Dominant Subdominant (DS) animals (4.5 ± 0.28) responded with significantly greater numbers of optimistic decisions compared to the SS (3.68 ± 0.22; p = 0.044) and S animals (2.77 ± 0.28; p = 0.002) (Fig 6A).

### Effects of space allocation and social class on the cognitive bias test

The Shapiro-Wilk test determined that these data were not normally distributed. A Kruskal-Wallis H test showed that there was no significant effect or interaction between cage size on the number of days featuring an optimistic decision χ^2^(1) = 0.044, p = 0.725. However, there was a statistically significant difference observed between social class on number of optimistic decisions made, χ^2^(1) = 5.865, p = 0.015. D animals responded with significantly greater number of optimistic decisions (453 ± 0.23) compared to S animals (3.63 ± 0.28; p = 0.015). (Fig 6B).

### Effects of housing density and social class on the social-interaction test

**Fig 7:**
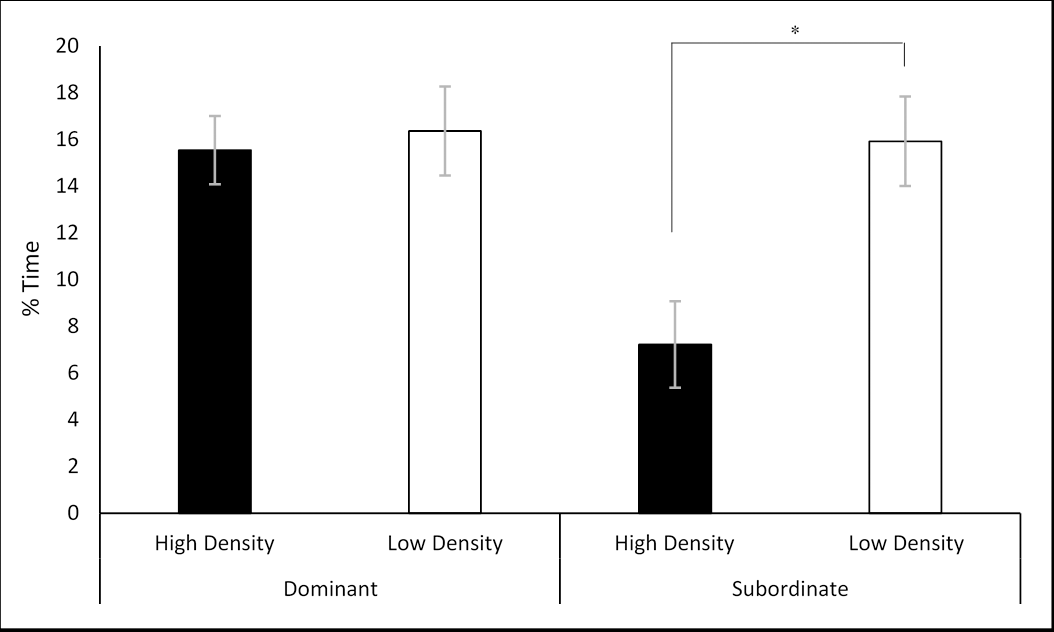
Interaction between density and class on the percentage time spent investigating the unfamiliar rat. Significance is denoted at p< 0.05.

A two-way MANOVA was performed fitting housing density and social class against the percentage of time spent exhibiting each of the scored behaviours. There was a statistically significant interaction between housing density and social class on the percentage of time the animal spent ‘investigating’ the unfamiliar, F (1, 57) = 6.878, p = 0.011. Simple main effects analysis showed that S rats housed in the high-density cages responded with a significantly reduced percentage of time investigating the unfamiliar rat (7.22% ± 1.91) compared to the S rats housed in the low-density cages (15.92% ± 1.85; p = 0.004) (Fig 7).

**Fig 8:**
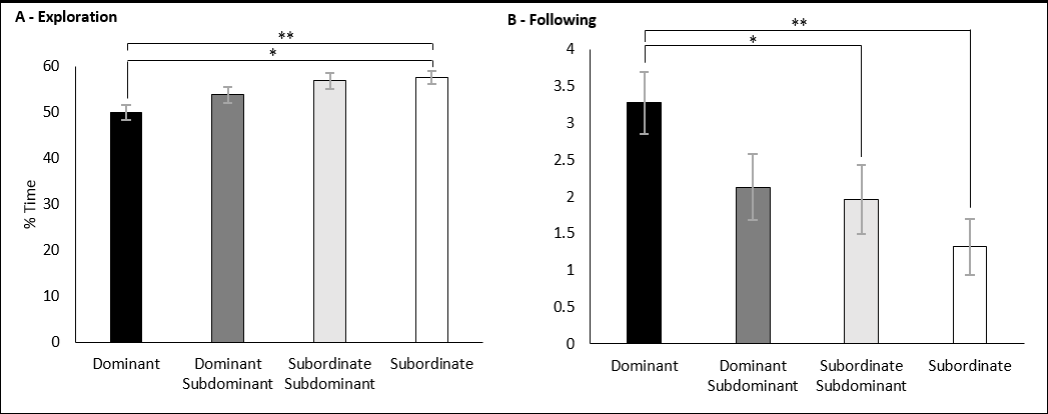
Effects of social class on the percentage of time spent exploring in the SIT. A) For the housing density experiment. B) For the space allocation experiment. Significance is denoted at p<0.05.

There was also a significant effect of social class on the percentage of time spent exploring the confines of the social-interaction test F (3,57) = 5.370, p = 0.003. D animals (49.98% ± 1.62) spent a significantly reduced percentage of time exploring the apparatus of the test when compared with both SS animals (56.81% ± 1.78; p = 0.004) and S animals (57.6% ± 1.44; p = 0.002) (Fig 8A). There was no significance detected for housing density on percentage time exploring, F (1,57) = 0.001, p = 0.992.

Finally, significance was also detected between social class and the percentage of time spent following the unfamiliar rat F (3,57) = 3.684, p = 0.017. D animals (3.27% ± 0.42) spent a significantly greater percentage of time following the unfamiliar rat then both SS animals (1.96% ± 0.43; p = 0.03) and S animals (1.37% ± 0.35; p = 0.002) (Fig 8B). There was no significance detected for housing density on percentage time following the unfamiliar rat, F (1,57) = 0.108, p = 0.744.

### Effects of space allocation and social class on the social-interaction test

**Fig 9:**
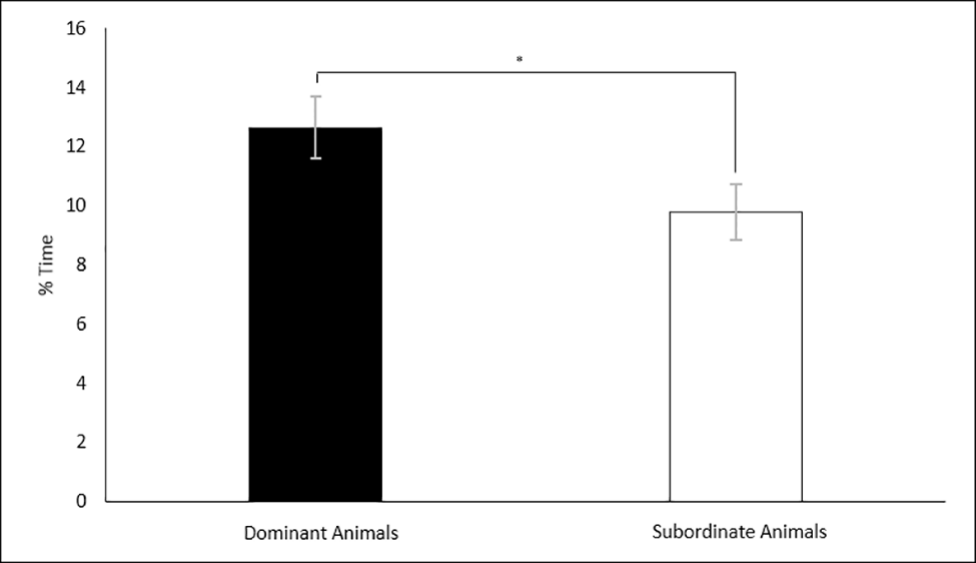
Effects of social class on the percentage of time spent exploring in the SIT. Results of the space allocation experiment. Significance is denoted at p < 0.05.

A two-way MANOVA was performed fitting space allocation and social class against the percentage of time spent exhibiting each of the scored behaviours. There were no statistically significant interactions between space allocation and social class on the percentage of time spent exhibiting any scored behaviour. However, there was a significant effect of social class on the percentage of time a rat spent investigating the unfamiliar rat. Dominant animals (12.64% ± 1.03) spent significantly greater percentages of time investigating the unfamiliar animals than the subordinate animals (9.79% ± 0.93) F (1, 22) = 4.301, p = 0.049 (Fig 9). No significance was detected between space allocation on the percentage of time investigating F (1, 22) = 0.572, p = 0.891.

**Fig 10:**
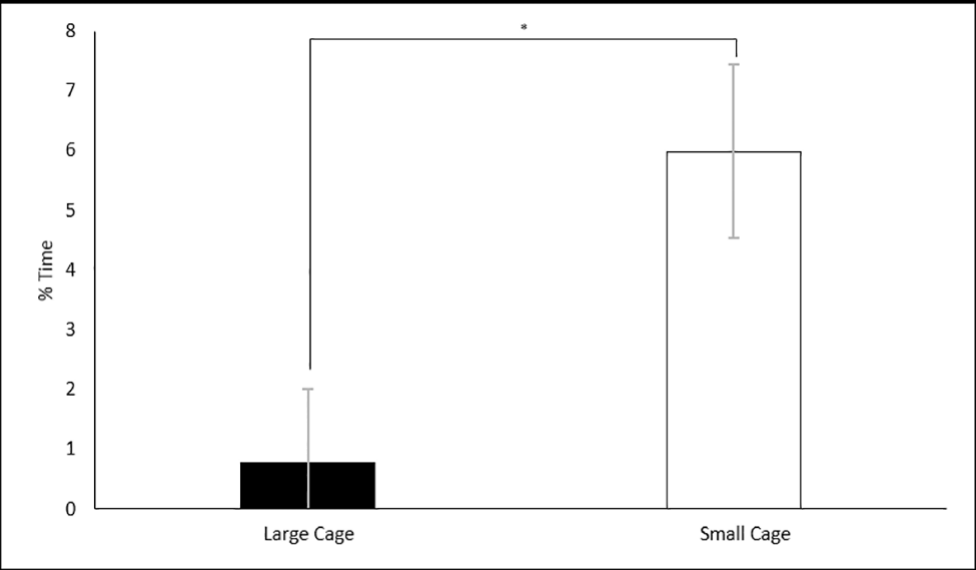
Effects of space allocation on the percentage of time following the unfamiliar in the SIT. Significance is denoted at p<0.05.

There was also a significant effect of space allocation on the percentage of time spent following the unfamiliar rat. Animals in the small cages (5.98% ± 1.45) spent a significantly greater percentage of time following the unfamiliar rat than compared to animals in the large cages (0.77% ± 1.23) F (1, 22) = 4.863, p = 0.038 (Fig 10). No significance was detected between social class on the percentage of time investigating F (1, 22) = 0.321, p = 0.613.

### Effects of housing density and social class on the novel-object recognition test

**Fig 11:**
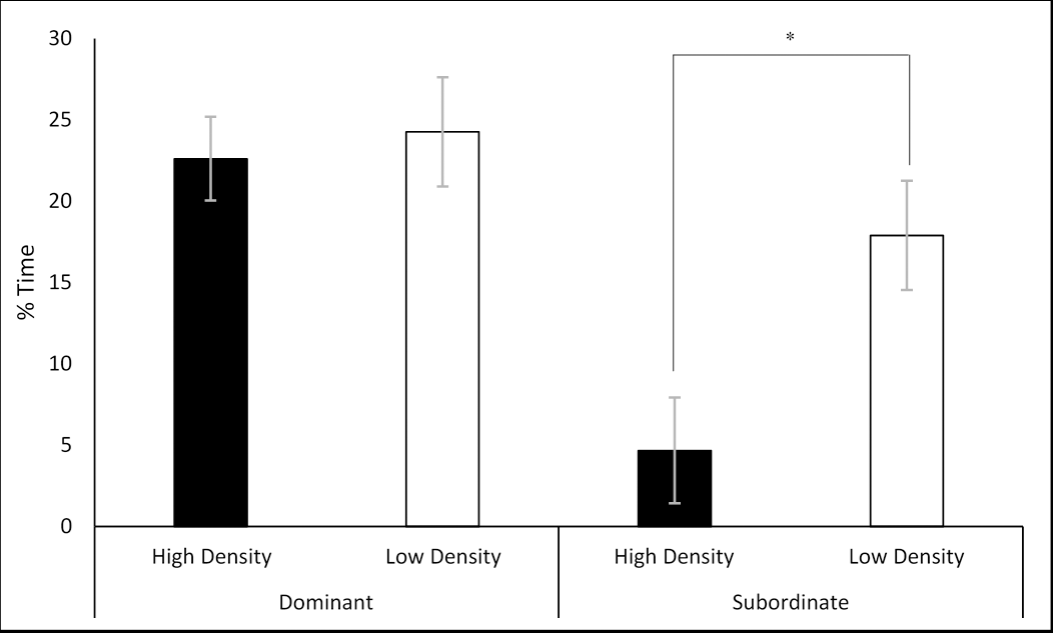
Interaction between housing density and class on percentage of time interacting with the novel object. Significance is denoted at p<0.05.

A two-way MANOVA was performed fitting housing density and social class against the percentage of time spent interacting with the familiar and the novel objects as well as the time spent exploring the cage parameters. There was a statistically significant interaction between housing density and social class on the percentage of time the animal spent interacting with the novel object F (1, 57) = 4.798, p = 0.033. Simple main effects analysis identified that S animals in the high-density cages responded with a significantly reduced percentage of time (4.673% ± 3.26) interacting with the novel object compared to S animals in the low-density cages (17.9% ± 3.36) (p = 0.012) (Fig 11).

### Effects of space allocation and social class on the novel-object recognition test

A two-way MANOVA was performed fitting space allocation and social class against the percentage of time spent interacting with the familiar and the novel objects as well as the time spent exploring the cage parameters. There was no statistically significant interaction or effect between these parameters on the test variables and therefore the data has been omitted.

### Effects of housing density and social class on the open-field test

**Fig 12:**
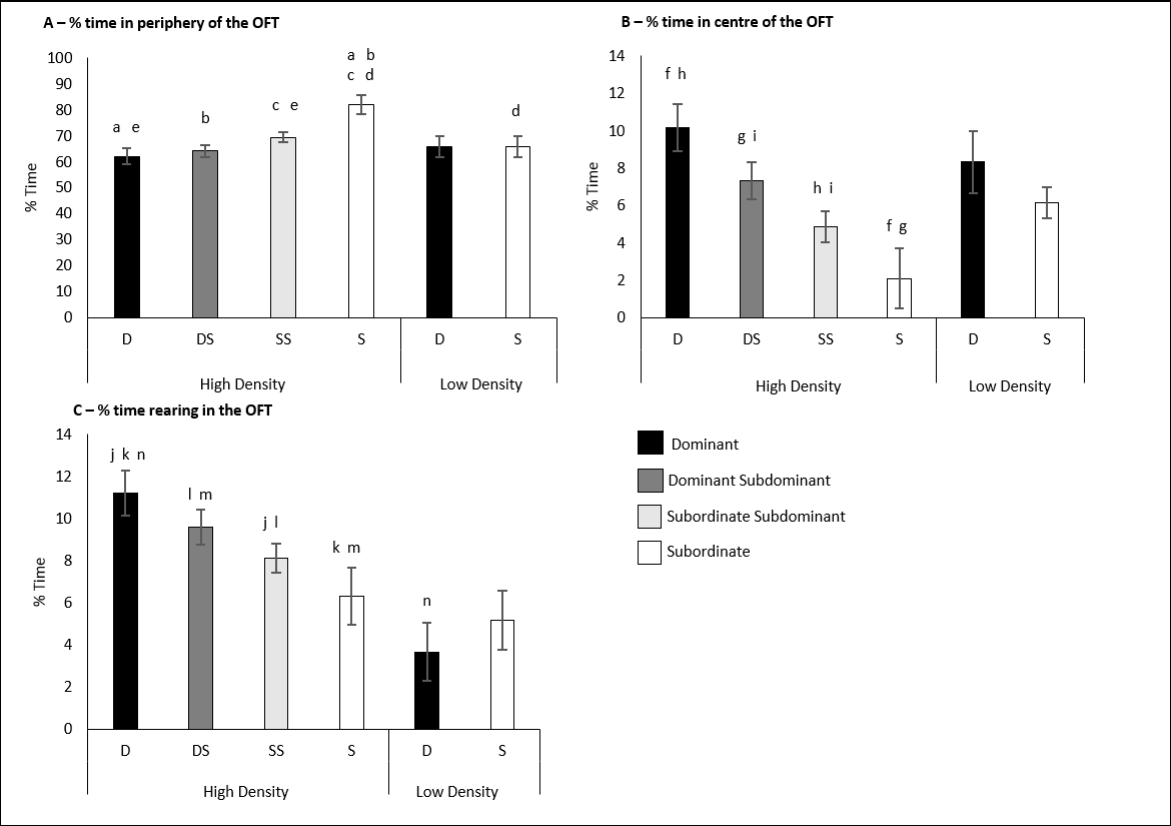
Results of the open field test for the housing density experiment. A) The interaction of housing density and social class on the percentage of time spent in the peripheral zone of the OFT. B) The interaction of housing density and social class on the percentage of time spent in the centre zone of the OFT. C) The interaction of housing density and social class on the percentage of time spent rearing in the OFT. Significance is denoted at p<0.05.

A two-way MANOVA was performed fitting housing density and social class against the percentage of time the animal spent in each of the open field testing ‘zones’ as well as the percentage of time spent defecating/urinating, rearing and being inactive. There was a significant interaction between housing density and social class on the percentage of time the animal spent in the peripheral zone of the OFT F (1,57) = 10.396, p = 0.002. Simple main effects analysis showed that S rats (81.99% ± 3.81) in the high-density cages responded with a significantly increased percentage of time in the peripheral zone compared to D rats (62.08% ± 3.01; p< 0.001), DS rats (64.1% ± 2.33; p< 0.001) and SS rats (69.43 ± 1.97; p = 0.003) also housed in the high-density cages. Likewise, S animals in the high-density cages also responded with increased percentage of time compared to S rats (65.82% ± 3.92; p = 0.009) housed in the low-density cages. SS rats of the high-density cages also responded with a significantly increased percentage of time in the peripheral zone compared to the D rats (p = 0.034) in the same cages. (Fig 12A). No significance was detected between social class for the low-density cages.

There was also a significant interaction between social class and housing density on the percentage of time the animals spent in the centre zone of the OFT, F (1,57) = 5.123, p = 0.027. S rats in the high-density cages (2.09% ± 1.6) responded with statistically significant decreases in the percentage of time spent in the centre compared to D rats (10.18% ± 1.27; p<0.001) and DS rats (7.34% ± 0.98; p = 0.005) of the high-density cages. Similarly, SS rats in the high-density cages (4.88% ± 0.83) also responded with a significantly reduced percentage of time in the centre zone compared to both D rats (p<0.001) and DS rats (p = 0.042) also in the high-density cages. Once again, there was no significance detected between social classes for the rats in the low-density cages (Fig 12B).

The final significant interaction between social class and housing density was between the percentage of time the animals spent rearing in the OFT, F (1,57) = 8.478, p = 0.005. S rats in the high-density cages (6.292% ± 1.35) once again responded with statistically significant decreases in the percentage of time spent rearing in the OFT compared to both the D rats (11.19% ± 1.07) (p<0.001) and the DS rats (9.57% ± 0.83) (p<0.001) also housed in high-density cages. SS rats in the high-density cages also responded with decreased percentage of time rearing compared to both D rats (p<0.001) and DS rat (p = 0.042). Finally, D rats housed in the low-density cages (3.66% ± 1.39) responded with a statistically significant decrease in the percentage of time rearing compared to D rats in the high-density cages (p<0.001). (Fig 12C).

### Effects of space allocation and social class on the open-field test

A two-way MANOVA was performed fitting space allocation and social class against the percentage of time the animal spent in each of the open field testing ‘zones’ as well as the percentage of time spent defecating/urinating, rearing and being inactive. The data for the percentage of time spent defecating/urinating was found to be non-parametric. Consequently, a Kruskal-Wallis H test was performed which revealed no significant effects or interactions between space allocation χ^2^(1) = 0.042, p = 0.838, and social class χ^2^(1) = 3.101, p = 0.078, on the percentage of time spent defecating/urinating in the open field test, and therefore this data has been omitted.

**Fig 13:**
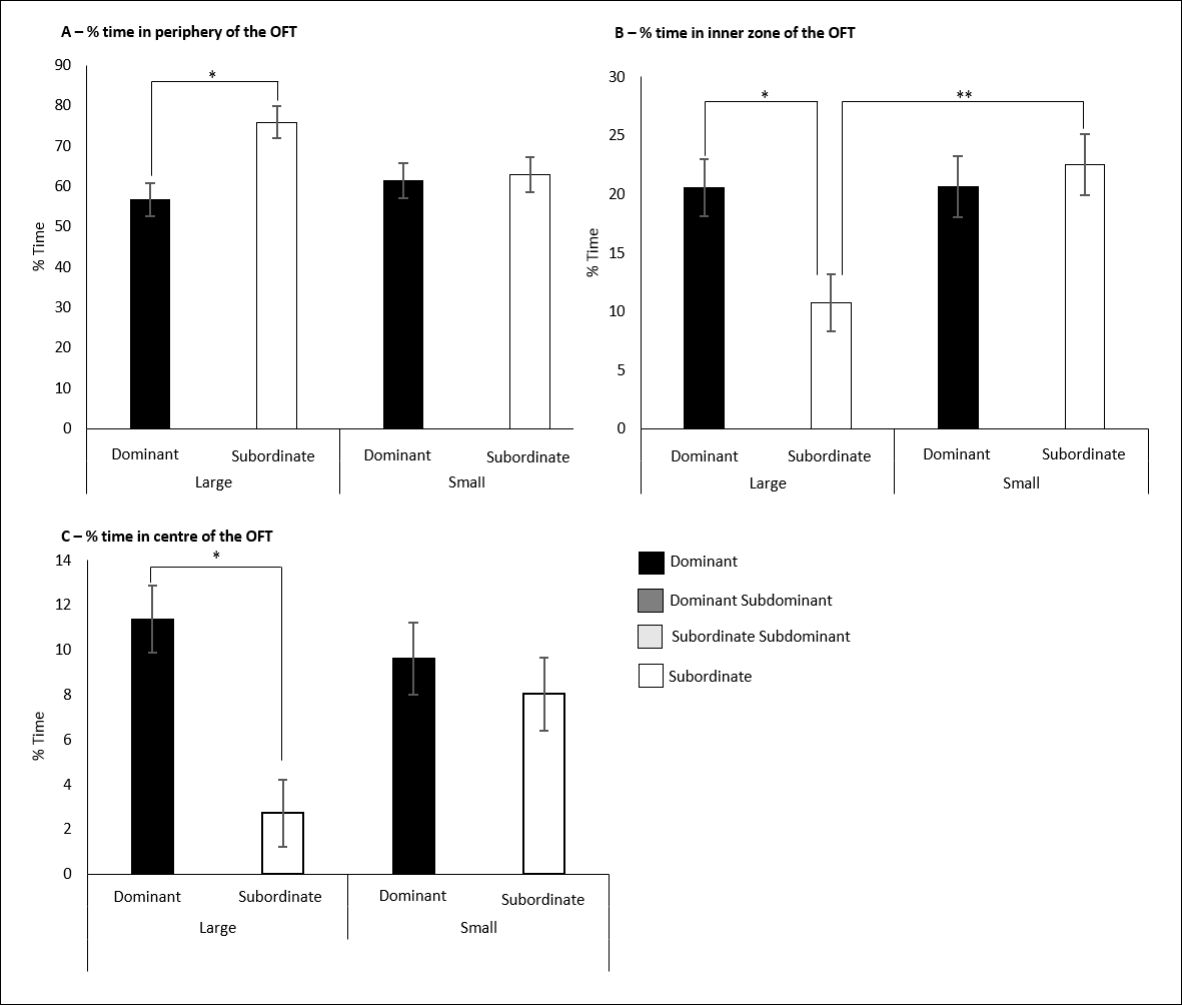
Results of the open field test for the space allocation experiment. A) The interaction of space allocation and social class on the percentage of time spent in the peripheral zone of the OFT. B) The interaction of space allocation and social class on the percentage of time spent in the inner zone of the OFT. C) The interaction of space allocation and social class on the percentage of time spent in the centre zone of the OFT. Significance is denoted at p< 0.05.

There was a significant interaction as observed from the two-way MANOVA of space allocation and social class on the percentage of time the rats spent in the peripheral zone, F (1,33) = 7.725, p = 0.009. Simple main effects analysis was employed to investigate this interaction. D rats in the large cages (56.74% ± 4.03) responded with a significantly reduced percentage of time in the peripheral zone compared to the S rats in the large cages (75.93% ± 4.03) (p<0.001) (Fig 13A).

There was another significant interaction between space allocation and social class on the percentage of time the rats spent in the inner zone, F (1,33) = 9.410, p = 0.004. Simple main effects analysis identified that S rats in the large cages (10.74% ± 2.42) responded with a significantly reduced percentage of time in the inner zone compared to D rats in the large cages (20.53% ± 2.41) (p = 0.001). S rats in the large cages also responded with a significantly reduced percentage compared to S rats in the small cages (22.53% ± 2.61) (p = 0.009) (Fig 13B).

The final significant interaction between space allocation and social class was on the percentage of time the animal spent in the centre zone, F (1,33) = 8.907, p = 0.005. Simple main effects analysis was used to investigate this interaction and showed that the S rats in the large cages (2.73% ± 1.5) responded with a significantly reduced percentage of time in the centre zone compared to the D rats in the large cages (11.36% ± 1.5) (p<0.001) (Fig 13C).

## Discussion

The study aimed to investigate the effects of housing density and space allocation on the behavioural performance of rats. No significant effects of housing density, and only one significant effect of space allocation was observed. There were several interactions between these factors and social status. Animal performance in the open-field [20], novel-object recognition [21] and social-interaction tests [18] are all highly repeated methods to observe anxiety-like behaviours in the rat. As hypothesised, subordinate animals regularly responded in these behavioural tests with significantly higher numbers of behaviours considered anxiety-like. Subordinate animals responded with significantly fewer optimistic decisions compared to their more highly ranked conspecifics. These significant effects and interactions indicated that cage size and social structure could potentially play a significantly greater role in the minimisation of social stressors than previously identified.

### Subordinate status is a source of anxiety in group-housed male rats

For both the housing density and space allocation experiments subordinate and subordinate-subdominant animals routinely responded with greater numbers of behaviours associated with an anxiogenic state. A reduced expression of optimistic cognitive biases (Fig 6A and 6B) has been associated with anxiety-like states in multiple animal species [13]. Subordinate animals demonstrated a decrease in the percentage of time spent following the unfamiliar rat in the SIT (Fig 8B) in both experiments, and a decreased percentage of time investigating the unfamiliar rat (Fig 9). A decrease in social investigatory behaviours has been reviewed and is associated with conditions known to cause anxiogenic responses [22]. Finally, a decrease in the amount of time an animal spent in the centre zones of the OFT (Fig 12B and 12C) was associated with anxiety and/or depression like conditions [20].

Subordination has been correlated with significant physiological changes, including decreases in bodyweight [23], reductions in dopamine activity of the brain [24], and teste weight, and decreased plasma testosterone and corticosterone levels [25]; changes consistent with chronic stress responses. Behavioural adaptions have also been associated with social class, with dominant animals spending significantly more time in the open arms of an elevated plus maze compared to subordinates [26]. Subordinate rats housed with a dominant conspecific also responded with an increase in the number of ultrasonic vocalisations (response to aversive stimuli) being emitted and displayed greater freezing responses than dominant conspecifics [27].

The results of the current study suggest that social status of rats can be a reliable cause of anxiety. Whilst there were several interactions of either housing density or space allocation with social status, there was no significant effect of housing density alone on any of the observed parameters and only one significant effect of space allocation (Fig 10). These interactions are discussed in more detail below. Investigations into housing density and space allocation on animal welfare are often confounded. The current study provides evidence that social status needs to be considered as an independent variable when studying the effects of either housing density, space allocation or the effects of crowding.

### Subordinate stress varies with housing density

The results suggested that the social stressors associated with subordination were not as severe in the low-density cages compared to the high-density cages. Subordinate animals of the high-density cages responded with a significantly decreased percentage of time investigating the unfamiliar rat in the SIT (Fig 7) and a decreased percentage of time interacting with the novel-object in the NORT (Fig 11). Reduced novel-object recognition is a sign of cognitive impairment in animals suffering from chronic stress [21]. In addition, subordinate animals of the high-density cages responded with significantly greater anxiety-like responses to the OFT (Fig 12A–12C) Previous study findings conducted in mice [28–30] and rats [10] support this observation in which aggression was intensified by increasing group size. In the current study, subordinate animals in the high-density cages experienced greater numbers of aggressive acts initiated upon them compared to the subordinates of the low-density cages. However, these discussed studies did not separate space allocation (surface area per animal) and housing density (animals per cage) this is discussed in greater detail later.

Studies that successfully decoupled space allocation from housing density have reported that aggressive encounters increased in larger groups of caged mice [31]. Another study reported that the number of aggressive encounters between the dominant and subordinate of highly dense cages (8 animals per cage) were significantly greater than animals housed in low density cages (3 animals per cage) [32]. A larger population size encourages the dominant animal to display greater levels of aggressive behaviour to sustain its dominant status. Meanwhile, subordinate animals of the high-density cages showed increased aggressive behaviours to possibly earn a higher social status within the hierarchy [32]. As summarised by Poole and Morgan [29] the greater the population size per cage, the more unstable the hierarchy, increasing the likelihood that dominance status would change between individuals.

Most previous studies have investigated mice, and given inherent species differences, it could be argued that these studies have limited relevance to the current study. However, many common behavioural tests of anxiety [33], learned helplessness [34] and general cognitive ability [35] report mouse and rat behaviour as being ‘equivalent’. This suggests that comparisons between the two species are valid when using tests to identify an anxiety or depressive like state. Therefore, while we cannot state that the subordinate stress experienced by the rats in the current study was equivalent to mice, or that the stress was caused in the same mechanistic manner, we can confidently state that the behavioural tests we employed to detect subordinate stress were appropriate.

The current study has shown that larger group sizes of rats lead to increases in the number of anxiety-like behaviours expressed by subordinate rats, and an overall increase in social stressors. This finding has been reported in mice, but has yet to be reported in rats. This encourages future research to focus on understanding the mechanisms underlying subordination stress in rats. Housing recommendations for lab animals are currently based on weight, with few guidelines based on experimental observations of appropriate group size. The findings of the current study should therefore be considered in future guidelines and legislative drafting.

### Subordinate stress varies with space allocation

As illustrated in Figs 6B and 13A–13C, subordinate animals in the large cages responded with significantly more anxiety-like behaviours compared to subordinate animals in the small cages. Furthermore, there were no significant differences observed at all, between dominant and subordinate animals in the small cages compared to those in the large cages. This suggested that subordinate stress was amplified with a larger area which the subordinate shared with the dominant. Guidelines and legislation tend to promote larger cages on the belief that an increased space allocation reduces crowding stress [3]. However, as discussed previously, this may stem from a failing of much peer-reviewed literature to successfully separate housing density and space allocation.

When provided with different cage sizes, rats would preferentially choose the larger cage (1620cm^2^ of usable floor space) that housed four other rats over a small cage (540cm^2^ of useable floor space), despite the larger cage providing less physical space for the rat to occupy than the small cage [36]. This suggested that rats preferentially chose conditions with greater crowding stressors than lone housing with a greater surface area allowance. Monogamous breeding pairs of Dahl salt-sensitive rats housed in small cages (922.6cm^2^ of useable floor space) showed no significant differences in breeding parameters compared to similar pairs housed in larger cages (1355cm^2^ of useable floor space) [37]. The 3^rd^ edition of the *Guide for the Care and Use of Agricultural Animals in Research and Testing* [38] states that reproductive parameters are an important indicator of animal welfare, suggesting that the use of smaller rat cages for breeding purposes is acceptable, despite it being considered ‘over-crowded’.

Crowding is often confounded with housing density, as an increase in housing density invariably leads to a crowding effect. Density has been defined as the number of animals occupying the same floor area. Crowding is defined as the motivational state that occurs when spatial and social factors interact, which influences behaviour to mitigate the effects of the restricted space [9]. The current study demonstrated that the larger the space allocation per animal, the greater the effect of the stressors associated with subordination. Van Loo, Mol [32] found both dominant and subordinate male mice housed in a small cage responded with fewer acts of aggression compared to those in larger cages. These authors concluded that a small cage was associated with dominant animals having a smaller defendable territory that reduced the number of aggressive acts needed to maintain control over this territory [32]. Therefore, it was hypothesised for the current study that the subordinate rats in the larger cages were subjected to more acts of aggression from their dominant cage-mates compared to the subordinate rats of the small cages.

This discussion highlights the need to consider the effects of crowding stress versus subordination stress, and future research should identify if the stressors from crowding produce more anxiety-like responses than subordination stress. Whilst increasing the surface area per animal will reduce the stressors associated with crowding, it may in turn increase the stressors associated with subordination.

### Social class and the effects of motivation in the judgment bias paradigm

Subordinate-subdominant and subordinate rats responded with fewer optimistic decisions then both the dominant and dominant-subdominant rats, when exposed to the JBP for both the housing density (Fig 6A) and space allocation experiments (Fig 6B). Likewise, these rats responded with increased time exploring the confines of the SIT (Fig 8A) and spent less time following the unfamiliar rat (Fig 8B) during the housing density experiment, signs previously associated with compromised sociability [39]. This suggested that an individual animal having a lower social rank not only encouraged pessimistic cognitive biases, but also encouraged social dissonance. A similar finding of sociability on cognitive bias expression reported that bottlenose dolphins that displayed more social affiliative behaviours (synchronous swimming) responded with greater numbers of optimistic decisions to a JBP [40]. This may provide an explanation as to why subordinate animals, which are the subjects of more acts of aggression and therefore fewer social affiliative behaviours, responded with significantly fewer optimistic decisions. We hypothesised that dominant animals experience a more harmonious social standing and therefore experience fewer anxiety-like effects associated with group housing. As discussed previously, large group sizes encourage animals of lower social standing to challenge other low ranked animals in order to gain a higher social status [32]. This finding is important as it suggests that animal status in a social hierarchy is an important covariate that needs to be taken into consideration when assessing animals on their cognitive bias expression using a JBP.

Dominance has also been correlated with an increased motivation for food reward, when housed in the visible burrow system (VBS), a model in which unfamiliar rats form dominance hierarchies. Dominant rats have been reported to respond to a palatable food reward with an increase in operant responses [26]. Therefore, it could be argued that a dominant social status in rats can significantly augment their ability to respond to palatable food rewards. Rats experiencing chronic social-stress have also shown reduced motivation-related behaviours. Using an indirect marker of dopamine activity (dopamine transporter binding density), non-responsive subordinate rats displayed long-lasting (3-week) changes in their mesolimbic dopaminergic system after experiencing a chronic-social stressor (VBS housing) [24]. This is significant when discussing the JBP as used in the current study, which relied on the motivating factor of food that encouraged the expression of a cognitive bias. Dominant animals have an innate increased motivation to attain a food reward, whilst subordinates experience physiological changes that decrease their ability to be ‘rewarded’. This implies that animals experiencing chronic-social stressors are less-motivated to perform reward-motivated behaviours, as they no longer receive “pleasure” by doing so.

If dominance status and subordination stress significantly alter behaviour [24] and food-motivation [26], then a food rewarded JBP to assess affective state of group-housed rats was a significant limitation of the study. Food-motivation has been established as a confounding variable when discussing animal behaviour in general [41, 42]. The possibility that coupling of food-motivation and social status acts as a confounding factor in the JBP is a novel theory. This suggestion renders many studies involving group-housed animals and a food-rewarded JBP confounded unless social status is included as a covariate in design. Logically, future work should then avoid a JBP that relies on animal motivation to food. This may prove difficult as the majority of previous JBP designs have utilised the presence or absence of food as the motivating factor [43]. Few JBP studies have reported success using location or social based rewards as the motivator to express a cognitive bias [44]. These tests may prove superior and should receive attention in future research.

## Conclusion

This study used an array of behavioural tests to explore the effects of housing density and space allocation on common laboratory rats. We were unable to confirm the hypothesis that increased housing density or a decreased space allocation would result in increased numbers of anxiety-like behaviours. However, we confirmed the hypothesis that subordinate rats would respond with greater anxiety-like behavioural traits compared to dominant rats. Furthermore, it was concluded that subordination stress in rats could be exacerbated by housing a greater number of rats in the same cage and by providing a greater surface area per animal. These findings are novel, being the first to successfully dissociate the commonly confused factors of space allocation and housing density in rats. Future work should include treatment groups of variable densities than those utilised in the current study, and a greater range of differently sized, commercially available cages.

Furthermore, the continued use of a reward-based JBP to assess the affective state in at least a group-housed rat model is discouraged. The combined factors of motivation and status within the social hierarchy can significantly augment behavioural expression of the rat. Future studies using a JBP in group-housed animal models should consider controlling for the social status of the animals.

This study has challenged the notion that rats have a greater standard of welfare when housed in larger cages, with more surface area per animal, a common presumption of rodent housing guidelines. An increased surface area does lead to a decrease in the negative effects associated with crowding. However, increasing the surface area also encourages the prevalence of anxiety-like behaviours associated with subordination. Therefore, simply increasing the surface area per rat may not lead to increased animal wellbeing. Furthermore, even when rats are housed with an approximately equivalent floor area per animal, those housed with more conspecifics experience greater levels of social stressors than those housed with a single cage-mate. Therefore, the data encourages the drafting of guidelines and legislative documents that do not simply increase the surface area of cages in which animals can be legally housed. Consideration of other factors such as cage complexity, housing density and social status will provide a higher standard of welfare for caged laboratory rats.

## Supporting information

S1 File Raw behavioural data used in the analysis. Data is presented as one excel spreadsheet. data has been separated into sheets by experiment (housing density or space allocation) and by behavioural test.
(XLSX)
